# Presence of hepatitis B virus in synovium and its clinical significance in rheumatoid arthritis

**DOI:** 10.1186/s13075-018-1623-y

**Published:** 2018-06-19

**Authors:** Yu-Lan Chen, Jun Jing, Ying-Qian Mo, Jian-Da Ma, Li-Juan Yang, Le-Feng Chen, Xiang Zhang, Tao Yan, Dong-Hui Zheng, Frank Pessler, Lie Dai

**Affiliations:** 1 Department of Rheumatology, Sun Yat-Sen Memorial Hospital, Sun Yat-Sen University, Guangzhou, People’s Republic of China; 2 Department of Radiology, Sun Yat-Sen Memorial Hospital, Sun Yat-Sen University, Guangzhou, People’s Republic of China; 3 Zhongshan School of Medicine, Sun Yat-sen University, Guangzhou, People’s Republic of China; 4 TWINCORE Center for Experimental and Clinical Infection Research, Hannover, Germany; 5 Helmholtz Center for Infection Research, Braunschweig, Germany

**Keywords:** Hepatitis B virus, Rheumatoid arthritis, Radiographic progression, Synovium, Synovial biopsy

## Abstract

**Background:**

Previous studies have revealed that hepatitis B virus (HBV) infection may be related to rheumatoid arthritis (RA), but there are no studies on the presence of HBV antigens or nucleic acid in synovium from patients with RA with HBV infection. In the present study, we investigated the presence of HBV in the synovium and its clinical significance in RA.

**Methods:**

Fifty-seven consecutive patients with active RA (Disease Activity Score 28-joint assessment based on C-reactive protein ≥ 2.6) and available synovial tissue who had completed 1 year of follow-up were recruited from a prospective cohort. The patients were divided into chronic HBV infection (CHB, *n* = 11) and non-CHB groups according to baseline HBV infection status. Clinical data were collected at baseline and at 1-, 3-, 6-, and 12-month follow-up. Radiographic changes of hand/wrist at baseline and month 12 were assessed with the Sharp/van der Heijde-modified Sharp score (mTSS). HBV in synovium was determined by immunohistochemical staining for hepatitis B virus surface antigen and hepatitis B virus core antigen (HBcAg) and by nested PCR for the HBV *S* gene.

**Results:**

HBcAg was found in the synovium of patients with RA with CHB (7 of 11, 64%), which was confirmed by PCR for the HBV *S* gene. Compared with the non-CHB group, more CD68-positive macrophages, CD20-positive B cells, and CD15-positive neutrophils infiltrated the synovium in the CHB group (all *p* <  0.05). There were smaller improvements from baseline in most disease activity indicators mainly at month 12, and a significantly higher percentage of CHB patients experienced 1-year radiographic progression (ΔmTSS ≥ 0.5 unit/yr, 64% vs. 26%, *p* = 0.024). Multivariate logistic regression analysis showed that CHB status (OR 14.230, 95% CI 2.213–95.388; *p* = 0.006) and the density of synovial CD68-positive macrophages (OR 1.002, 95% CI 1.001–1.003; *p* = 0.003) were independently associated with 1-year radiographic progression.

**Conclusions:**

The presence of HBV in RA synovium may be involved in the pathogenesis of local lesions and exacerbate disease progression in RA.

**Electronic supplementary material:**

The online version of this article (10.1186/s13075-018-1623-y) contains supplementary material, which is available to authorized users.

## Background

Rheumatoid arthritis (RA) is a chronic systemic autoimmune disorder characterized by synovitis and bone/cartilage destruction [1]. Even though the etiology of RA remains unknown, there is evidence that it results from a combination of genetic predisposition and environmental factors, especially infectious agents such as Epstein-Barr virus, cytomegalovirus, and *Proteus mirabilis* [2]. Molecular mimicry on the basis of amino acid similarities shared by viral and self-antigens has long been proposed as a pathogenic mechanism for RA [3]. However, the pathogenicity of other infectious agents linked to RA remains to be identified.

Hepatitis B virus (HBV) infection is a major cause of chronic liver diseases, such as liver cirrhosis and hepatocellular carcinoma. The genome of this DNA virus encompasses four partially overlapping open reading frames, of which the *pre-S/S* region encodes the viral surface antigen (HBsAg) and the *pre-core/core* gene encodes the e antigen (HBeAg) and the core antigen (HBcAg) [4]. HBV infects not only human hepatocytes but also diverse extrahepatic tissues such as lymph nodes, kidney, skin, colon, stomach and pancreas, which leads to extrahepatic manifestations in patients with HBV infection or HBV-related diseases such as glomerulonephritis and polyarteritis nodosa [5]. Some patients with symptoms induced by recombinant hepatitis B vaccination were reported to fulfil the 1987 revised criteria of the American College of Rheumatology (ACR) for RA and required disease-modifying anti-rheumatic drug (DMARD) therapy [6]. The serum HBsAg positivity reported in our previous study was 11.2% in Chinese patients with RA, compared with 8.7% in the age-matched Chinese general population [7]. In agreement with this, a recent study revealed a higher HBV period prevalence in 38,969 patients with RA than in 701,476 non-RA control subjects in Taiwan [8], which further supported the hypothesis that HBV infection has a subtle association with RA. In the 1970s, Schumacher et al. first reported the presence of HBV in the synovium of two patients with arthritis with HBV infection using direct immunofluorescence, and they also found virus particles mainly in synovial lining cells and vascular endothelium by electron microscopy [9]. A case report published in 2006 described a patient with knee osteoarthritis who had positive serum HBsAg and HBeAg experienced rapidly destructive knee arthropathy; in that report, immunohistochemical staining revealed diffuse HBsAg expression in the patient’s synovium [10]. Nevertheless, no study regarding the presence of HBV in the synovium from patients with RA has been reported. The aim of this study was to investigate the frequency of HBV infection in the synovium of patients with coexisting RA and to determine its influence on histopathological characteristics of synovitis as well as clinical and radiographic outcomes in RA.

## Methods

### Study patients

Consecutive patients with RA who fulfilled the 1987 ACR revised criteria [11] or the 2010 ACR/European League Against Rheumatism (EULAR) criteria [12] for RA classification were retrospectively recruited from a prospective RA cohort (*n* = 239) in the Department of Rheumatology at Sun Yat-Sen Memorial Hospital from June 2013 to August 2016. The inclusion criteria in this study also included the following: active disease, defined as the Disease Activity Score in 28 joints with four variables including C-reactive protein (DAS28-CRP) ≥ 2.6; availability of synovial tissue at baseline passing quality criteria (at least six pieces containing lining layer and sublining area to a depth of at least 1 high-power microscopic field, 400× magnification); and completion of at least 1 year of follow-up. The exclusion criteria were as follows: overlap with other autoimmune diseases (e.g., systemic lupus erythematosus, scleroderma, dermatomyositis, polyarteritis nodosa); presence of liver cirrhosis or hepatocellular carcinoma, Wilson’s disease, steatohepatitis, hemochromatosis, or schistosomiasis japonica; concomitant infection with hepatitis C virus, hepatitis D virus, human immunodeficiency virus, or other serious infection, organ dysfunction, or malignancy; and being lactating, pregnant, or planning to become pregnant. All participants gave their written informed consent before clinical data collection. The study was approved by the Medical Ethics Committee of Sun Yat-Sen Memorial Hospital (identifier SYSEC-2009-06).

### Serology and virology of HBV infection and patient grouping

Serological markers of HBV infection, including HBsAg, antibodies to hepatitis B surface antigen (anti-HBs), HBeAg, antibodies to hepatitis B e antigen (anti-HBe), and antibodies to hepatitis B core antigen (anti-HBc), were tested in all patients with RA by electrochemiluminescence immunoassay (Roche Diagnostics, Mannheim, Germany). Serum HBV DNA level was measured with a commercially available qRT-PCR kit (Da An Gene Co., Ltd. of Sun Yat-Sen University, Guangdong, China), with a limit of detection of 500 IU/ml. The diagnosis of HBV infection fulfilled the Chinese guidelines for prevention and treatment of chronic hepatitis B [13]. Chronic hepatitis B virus infection (CHB) was defined as positive HBsAg and (or) HBV DNA persisting in serum for ≥ 6 months. Resolved HBV infection was defined as negative HBsAg and HBV DNA in serum but positive anti-HBc. Non-HBV infection was defined as negative HBsAg, HBeAg, anti-HBe, anti-HBc, and HBV DNA in serum, regardless of anti-HBs status. According to the baseline HBV infection status, all patients were divided into a CHB group and a non-CHB group (resolved HBV and non-HBV).

### Clinical data collection

Demographic and clinical data were collected at baseline and at 1-, 3-, 6-, and 12-month follow-up as in our previous report and modified according to the 2017 EULAR recommendations [14], including the 28-joint tender and swollen joint count (28TJC and 28SJC, respectively), patient and provider global assessment of disease activity (PtGA and PrGA, respectively), pain visual analogue scale (Pain VAS), the Stanford Health Assessment Questionnaire Disability Index (HAQ-DI), erythrocyte sedimentation rate (ESR), C-reactive protein (CRP), serum rheumatoid factor (RF), and anti-cyclic citrullinated peptide antibody (ACPA). Disease activity was assessed with DAS28-CRP, the Disease Activity Score in 28 joints with four variables including ESR (DAS28-ESR), the Simplified Disease Activity Index (SDAI), the Clinical Disease Activity Index (CDAI), and the Routine Assessment of Patient Index Data 3 (RAPID3).

HBV serological markers and HBV DNA levels were evaluated in all patients with RA at baseline and every 1–3 months during follow-up in the CHB group. These parameters in the non-CHB group were reexamined if aminotransferase activity was elevated during follow-up. Liver function, including alanine aminotransferase (ALT, U/L, normal range 5–40 U/L) and aspartate transaminase (AST, U/L, normal range 5–40 U/L), as well as bilirubin as clinically indicated, was also tested at each visit.

### Radiographic assessments

Radiographic assessments of bilateral hands and wrists (anteroposterior view) were done at baseline and month 12. Joint damage, including joint erosion (JE) and joint space narrowing (JSN), was assessed with the Sharp/van der Heijde modified Sharp score (mTSS) by two experienced observers (JDM from the Department of Rheumatology and XZ from the Department of Radiology) who were blinded to clinical data as we described previously [15]. Reliability and agreement were assessed using an intraclass correlation coefficient (ICC): the mean ICC for interobserver agreement was 0.90. Bony erosion was defined when a cortical break was detected by radiography [16]. Radiographic progression was defined as a change of mTSS (ΔmTSS) ≥ 0.5 unit after 1 year [17]. Rapid radiographic progression was defined as ΔmTSS ≥ 5 units after 1 year [18].

### Immunohistochemical and synovitis assessments

All synovial tissues in this study were obtained by closed Parker-Pearson needle biopsy from actively inflamed knee joints of patients with RA [19, 20]. Samples were fixed in 10% neutral formalin and embedded in paraffin. Serial sections of synovium (3 μm thick) were stained with H&E and immunohistochemically stained according to a three-step immunoperoxidase method. Sections were stained with anti-human HBsAg (Novocastra Laboratories Ltd., Newcastle Upon Tyne, UK; and Maixin Biotechnologies Ltd., Fuzhou, Fujian, China), HBcAg (Dako, Carpinteria, CA, USA), and the following commercial antibody preparations (Life Technologies, Carlsbad, CA, USA; and Novocastra Laboratories Ltd., Newcastle Upon Tyne, UK) according to standard staining protocols: anti-CD20 (clone L26, B cells), anti-CD38 (clone SPC32, plasma cells), anti-CD3 (clone PS1, T cells), anti-CD68 (clone KP1, macrophages), anti-CD15 (clone My1, neutrophils), and anti-CD34 (clone QB End/10, vascular endothelial cells). All antibodies were mouse monoclonal antibodies, except anti-HBcAg (rabbit polyclonal antibodies). Parallel sections were incubated with irrelevant, isotype, and concentration-matched monoclonal antibodies as a negative control, and liver tissues from patients with HBV-related hepatocellular carcinoma were used as a positive control. Histopathological changes in H&E-stained sections were graded according to the Krenn synovitis score [21–23]. The densities of cells with positive staining for CD3, CD15, CD20, CD38, and CD68 and the microvessel count (MVC; confirmed by the presence of CD34-positive endothelial cells in vessels with diameter ≤ 8 erythrocytes) were determined using manual counting by two independent trained investigators (LFC from the Department of Rheumatology and TY from Zhongshan School of Medicine) who were blinded to the clinical data. The densities are given as cells per square millimeter [20, 24].

### Detection of HBV DNA in the synovium

The HBV *S* gene was detected by nested PCR as described previously [25]. HBV DNA was extracted from about 30 mg (obtained from approximately 20 sections, 5 μm thick, not attached to glass slides) of paraffin-embedded synovium with the RecoverAll™ total nucleic acid isolation kit (Life Technologies). Liver tissue from patients with HBV-related hepatocellular carcinoma was included as a positive control. Amplification was carried out in a 50-μl reaction volume containing 3 μl of forward and reverse primers (10 μM), 40 ng of DNA template, and 25 μl of 2 × KAPA HiFi HotStart ReadyMix (Kapa Biosystems, Wilmington, MA, USA). The following thermocycles were used: 95 °C for 3 minutes, followed by 35 cycles of 98 °C for 20 seconds, 65 °C for 15 seconds, and 72 °C for 1 minute, with a final extension at 72 °C for 1 minute. The PCR products were then resolved by gel electrophoresis (Life Technologies). DNA bands were visualized by ultraviolet fluorescence. PCR products were sequenced in both directions on an ABI 3730 XL Automated DNA Sequencer with the ABI BigDye Terminator v3.1 cycle sequencing kit (Applied Biosystems, Foster City, CA, USA). The sequences were aligned using the Basic Local Alignment Search Tool (National Center for Biotechnology Information website https://blast.ncbi.nlm.nih.gov/Blast.cgi) to confirm the identity of the HBV *S* gene.

### Statistical analysis

IBM SPSS Statistics 20.0 for Windows software (IBM, Armonk, NY, USA) was used for statistical analyses. For continuous variables, the Mann-Whitney *U* test or Kruskal-Wallis analysis of variance on ranks between two groups or among three groups was used, and descriptive statistics (median, interquartile range (IQR)) were calculated. The Wilcoxon matched-pairs signed-rank sum test was used to compare the differences of continuous variables between disease activity indicators at baseline and each visit. For categorical variables, the Chi-square test or Fisher’s exact test was used, and indicators are presented as frequencies and percentages. Spearman’s rank-order correlation test was used to assess the relationship between serum levels of HBV DNA and RA disease characteristics in the CHB group. Logistic regression analyses were performed to identify risk factors for 1-year radiological progression by adjusting for confounding factors. Variables were included in the equation when *p* <  0.05 or removed when *p* > 0.10 following the stepwise forward selection rule. A two-tailed *p* <  0.05 was considered statistically significant.

## Results

### Baseline characteristics

Baseline characteristics of the 57 included patients with RA are shown in Table 1. There were 43 (75%) female patients. The median age of all patients was 51 years and the median disease duration was 24 months. Eighty-four percent of patients had bony erosion at baseline, and 61% of the patients were without glucocorticosteroid or DMARD therapy in the 6 months before entry into the study (treatment-naïve). According to HBV infection status at baseline, there were 11 (19%), 22 (39%), and 24 (42%) patients with CHB, resolved HBV, and non-HBV infection, respectively. In the CHB group, eight patients had detectable serum HBV DNA at baseline, ranging from 5.00 × 10^2^ IU/ml to 6.96 × 10^7^ IU/ml; four patients had positive HBeAg, but only one had abnormal liver function (AST 50 U/L and ALT 66 U/L). All CHB patients showed persistently positive HBsAg in serum during the 1-year follow-up.

**Table 1:** Baseline characteristics of patients

Parameters	All patients (*n* = 57)	CHB group (*n* = 11)	Non-CHB group (*n* = 46)	*p* Value^a^
Demographic characteristics				
Female, *n* (%)	43 (75)	7 (64)	36 (78)	0.311
Age, yr	51 (45–59)	49 (41–53)	52 (46–60)	0.311
Disease duration, mo	24 (7–102)	120 (6–120)	24 (7–72)	0.345
Smoking, *n* (%)	12 (21)	2 (18)	10 (22)	0.795
Disease activity indicators				
TJC28	9 (5–16)	5 (2–9)	11 (5–16)	**0.021**
SJC28	6 (3–9)	5 (2–8)	6 (4–10)	0.300
PtGA	6 (5–8)	5 (3–6)	6 (5–8)	**0.007**
PrGA	6 (4–7)	5 (3–6)	6 (5–8)	**0.006**
Pain VAS	5 (4–6)	4 (2–5)	6 (4–7)	**0.023**
CRP, mg/L	32.0 (14.8–61.0)	24.9 (15.4–66.8)	32.6 (14.2–55.6)	0.656
ESR, mm/h	68 (45–98)	63 (37–105)	70 (49–93)	0.627
Positive RF, *n* (%)	49 (86)	10 (91)	39 (85)	0.599
Positive ACPA, *n* (%)	52 (91)	11 (100)	41 (89)	0.252
DAS28-CRP	5.3 (4.6–6.1)	4.7 (4.2–5.8)	5.6 (5.0–6.3)	**0.024**
DAS28-ESR	6.2 (5.4–7.0)	5.4 (4.7–6.2)	6.4 (5.6–7.0)	**0.019**
SDAI	31.8 (21.8–42.3)	22.1 (17.5–34.2)	33.0 (26.9–43.7)	**0.026**
CDAI	27 (19–39)	19 (10–27)	30 (22–41)	**0.009**
RAPID3	12.7 (8.8–14.8)	7.1 (5.5–12.4)	13.1 (10.6–15.1)	**0.001**
HAQ-DI	1.3 (0.6–1.9)	0.5 (0.1–1.4)	1.4 (0.9–2.0)	**0.014**
Liver function				
AST, U/L	16 (14–23)	22 (17–28)	16 (14–18)	**0.027**
ALT, U/L	15 (11–21)	19 (14–31)	15 (11–19)	0.087
Radiographic status				
Bony erosions, *n* (%)	48 (84)	9 (82)	39 (85)	0.809
JSN subscore	4 (0–17)	1 (0–11)	5 (1–18)	0.331
JE subscore	7 (2–20)	2 (1–23)	9 (2–19)	0.447
mTSS	12 (4–34)	9 (1–34)	13 (4–35)	0.352
Previous medications, *n* (%)				
Treatment-naïve^b^	35 (61)	5 (46)	30 (65)	0.226
Glucocorticosteroids	21 (37)	4 (36)	17 (37)	0.971
Methotrexate	10 (18)	3 (27)	7 (15)	0.345
Leflunomide	10 (18)	0	10 (22)	NA
Sulfasalazine	6 (11)	2 (18)	4 (9)	0.357
Hydroxychloroquine	5 (9)	3 (27)	2 (4)	**0.016**
Biologic DMARDs	1 (2)	0	1 (2)	NA
Initial medications, *n* (%)				
Glucocorticosteroids	44 (77)	8 (73)	36 (78)	0.694
Methotrexate	54 (95)	10 (91)	44 (96)	0.527
Leflunomide	39 (68)	0	39 (85)	**< 0.001**
Sulfasalazine	10 (18)	9 (82)	1 (2)	**< 0.001**
Hydroxychloroquine	12 (21)	10 (91)	2 (4)	**< 0.001**
Biologic DMARDs	25 (44)	3 (27)	22 (48)	0.217

*Abbreviations: ACPA* Anti-cyclic citrullinated peptide antibody, *ALT* Alanine aminotransferase, *AST* Aspartate transaminase, *CDAI* Clinical Disease Activity Index, *CHB* Chronic hepatitis B virus infection, *CRP* C-reactive protein, *DAS28* Disease Activity Score 28-joint assessment, *DMARD* Disease-modifying anti-rheumatic drug, *ESR* Erythrocyte sedimentation rate, *HAQ-DI* Stanford Health Assessment Questionnaire Disability Index, *JE* Joint erosion, *JSN* Joint space narrowing, *mTSS* Modified total Sharp score, *NA* Not applicable, *Pain VAS* Pain visual analogue scale, *PrGA* Provider global assessment of disease activity, *PtGA* Patient global assessment of disease activity, *RA* Rheumatoid arthritis, *RAPID3* Routine Assessment of Patient Index Data 3, *RF* Rheumatoid factor, *SDAI* Simplified Disease Activity Index, *SJC28* 28-joint swollen joint count, *TJC28* 28-joint tender joint count

^a^Comparison between the CHB and non-CHB groups. Data correspond to number (percent) or median (interquartile range) unless stated otherwise. Bold *p* values indicate statistically significant levels

^b^Without glucocorticosteroid or disease-modifying anti-rheumatic drug therapy in the 6 months before entry into the study

### HBV detection in synovium

**Fig. 1:**
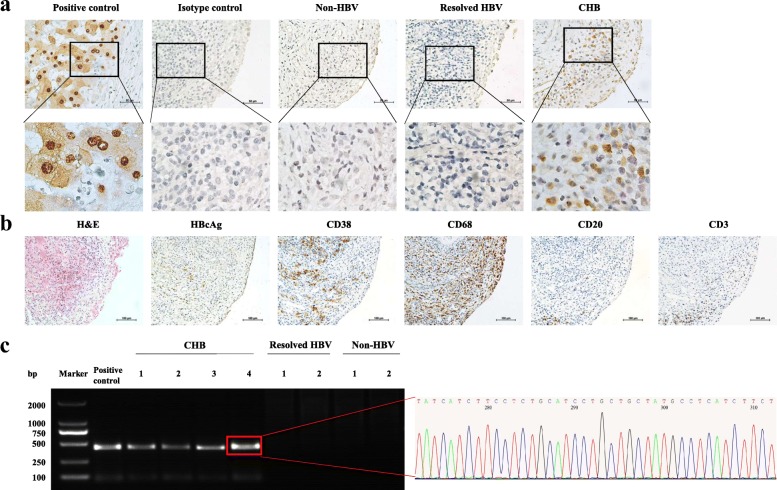
Identification of hepatitis B virus (HBV) in rheumatoid arthritis (RA) synovium. **a** and **b** Immunohistochemical staining for hepatitis B virus core antigen (HBcAg) in RA synovium. Representative images illustrate detection of HBcAg in patients with RA with chronic hepatitis B virus infection (CHB). HBcAg immunoreactivity was observed in sublining plasma cells (CD38^+^) and macrophages (CD68^+^), mainly located in the cytoplasm. **c** Detection of the HBV *S* gene in RA synovium by nested PCR and DNA sequencing. The HBV *S* gene was detected exclusively in the four CHB synovial tissue samples. Liver tissue from a patient with HBV-related hepatocellular carcinoma was used as positive control

The results of immunohistochemical staining of synovial tissue were negative for HBsAg in all samples from both the CHB and the non-CHB groups, but they were positive for HBcAg in synovial tissues from seven CHB patients, of whom five had detectable serum HBV DNA (ranging from 5.00 × 10^2^ IU/ml to 6.96 × 10^7^ IU/ml) and two had positive serum HBeAg. HBcAg immunoreactivity was observed in CD38-positive plasma cells and CD68-positive macrophages in the sublining area, located mainly in the cytoplasm (Fig. 1a and b). HBcAg was not detected in the other four patients with CHB or in the non-CHB patients.

The presence of the HBV *S* gene was tested by nested PCR in synovial tissue from four patients with CHB with positive synovial HBcAg immunoreactivity, two patients with resolved HBV infection, and two non-HBV patients. The HBV *S* gene was detected only in the four CHB samples (Fig. 1c). Further DNA sequencing of PCR products confirmed the specificity of amplification, demonstrating the presence of HBV DNA in the synovium from patients with RA and CHB.

### HBV infection and histopathological synovitis

Synovial histopathological features were compared in patients with RA with and without CHB (Table 2). Compared with non-CHB synovium, significantly higher densities of total and sublining CD68-positive macrophages, CD20-positive B cells, and CD15-positive neutrophils were observed in the CHB specimens (all *p* **<** 0.05). Further comparison was performed between CHB synovium with and without positive HBcAg. Remarkably, compared with CHB synovium with negative HBcAg (*n* = 4), there were significantly more MVC, sublining CD68-positive macrophages and CD20-positive B cells infiltrating the CHB synovium with positive HBcAg (*n* = 7), with a higher subscore of synovial stroma activation (all *p* **<** 0.05).

**Table 2:** Comparison of synovial histopathological features

Parameters	CHB group^a^ (*n* = 11)	Non-CHB group^a^ (*n* = 46)	*p* Value^b^	HBcAg(+)^c^ (*n* = 7)	HBcAg(−)^c^ (*n* = 4)	*p* Value^d^
MVCs, /mm^2^	145 (102–216)	140 (118–177)	0.473	133–286	93–145	**0.023**
CD3^+^ T cells, /mm^2^	1141 (560–1751)	639 (473–1131)	0.124	0–2103	425–1934	0.571
CD15^+^ neutrophils, /mm^2^	638 (297–897)	229 (149–389)	**0.010**	63–1367	16–1484	0.850
CD20^+^ B cells, /mm^2^	1216 (472–2834)	340 (122–753)	**0.001**	773–4695	267–1940	**0.038**
CD38^+^ plasma cells, /mm^2^	1594 (380–2223)	815 (269–1346)	0.124	519–4486	120–1795	0.059
CD68^+^ macrophages^e^, /mm^2^	1873 (1016–2304)	923 (622–1310)	**< 0.001**	1016–2806	968–2826	0.257
Sublining CD68^+^ macrophages	1686 (871–2075)	659 (449–1005)	**< 0.001**	991–2496	784–1345	**0.023**
Lining CD68^+^ macrophages	202 (158–238)	212 (126–266)	0.911	158–330	193–480	0.450
Krenn synovitis score	4 (2–7)	5 (4–6)	0.682	2–7	1–4	0.071
Hyperplasia of lining layer	2 (1–2)	2 (1–2)	0.808	1–3	1–2	0.308
Inflammatory infiltration	1 (1–2)	1 (1–2)	0.514	0–3	0–1	0.072
Synovial stroma activation	1 (1–2)	2 (1–2)	0.278	1–3	0–1	**0.025**

*Abbreviations: CHB* Chronic HBV infection, *HBcAg* Hepatitis B virus core antigen, *MVC* Microvessel count, *RA* Rheumatoid arthritis

^a^Data correspond to median (interquartile range) unless stated otherwise. Bold *p* values indicate statistically significant levels

^b^Comparison between the CHB and non-CHB groups

^c^Data correspond to minimum - maximum

^d^Comparison of HBcAg-positive vs. HBcAg-negative synovial specimens from patients with CHB

^e^CD68-positive macrophages included lining and sublining CD68-positive macrophages

### Clinical responses

**Fig. 2:**
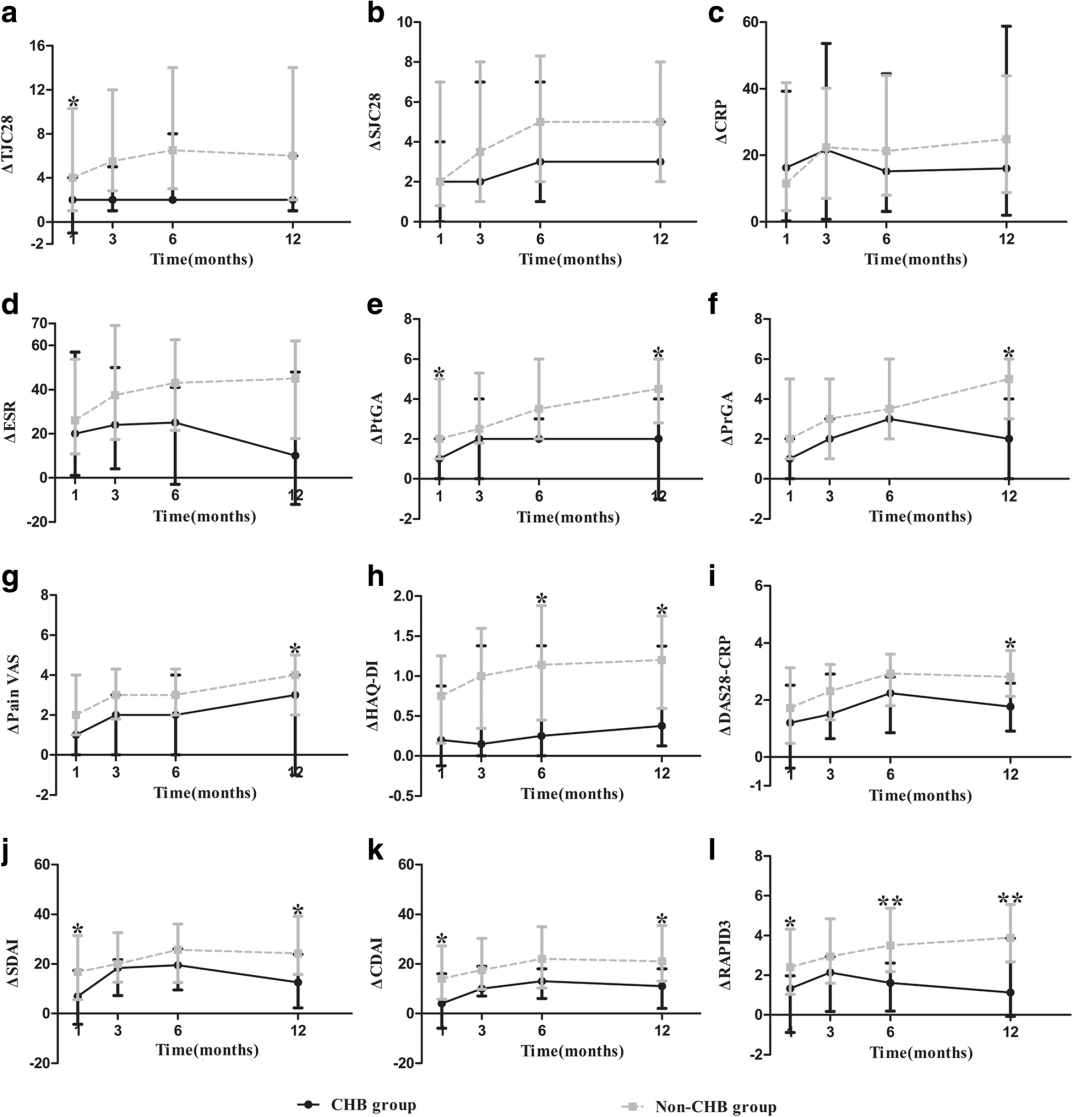
Comparison of improvements in disease activity indicators between patients with rheumatoid arthritis (RA) with and without chronic hepatitis B virus infection (CHB). **a–l** Compared with the non-CHB group, patients with RA in the CHB group experienced significantly smaller improvements from baseline in most disease activity indicators mainly at month 12 (including PtGA, PrGA, Pain VAS, HAQ-DI, DAS28-CRP, SDAI, and CDAI), in TJC28 at month 1, and especially RAPID3 at almost each point except month 3, but no significant improvements were observed in SJC28, CRP, or ESR. *p < 0.05, **p < 0.01. *CDAI* Clinical Disease Activity Index, *CRP* C-reactive protein, *DAS28* Disease Activity Score 28-joint assessment, *ESR* Erythrocyte sedimentation rate, *HAQ-DI* Stanford Health Assessment Questionnaire Disability Index, *Pain VAS* Pain visual analogue scale, *PrGA* Provider global assessment of disease activity, *PtGA* Patient global assessment of disease activity, *RAPID3* Routine Assessment of Patient Index Data 3, *SDAI* Simplified disease activity index, *SJC28* 28-joint swollen joint count, *TJC28* 28-joint tender joint count

All patients were treated according to the “treat-to-target” strategy, the patient’s willingness, and the patient’s HBV infection status [26, 27]. Ten (91%) patients in the CHB group accepted antiviral prophylaxis, including entecavir (*n* = 4), lamivudine (*n* = 4), and adefovir (*n* = 2). Compared with the non-CHB group, patients with RA with CHB showed significantly lower levels of most disease activity indicators at baseline, including TJC28, PtGA, PrGA, Pain VAS, DAS28-CRP, DAS28-ESR, SDAI, CDAI, and RAPID3 (all *p* <  0.05) (Table 1). During 1-year follow-up, significant improvement from baseline was observed in disease activity indicators at each visit (all *p* <  0.001) (Additional file 1). However, compared with the non-CHB group, patients with RA with CHB experienced smaller improvements from baseline in most disease activity indicators, mainly at month 12, including PtGA, PrGA, Pain VAS, HAQ-DI, DAS28-CRP, SDAI, and CDAI, especially RAPID3 at almost each point except month 3 (all *p* <  0.05) (Fig. 2). There were no significant differences in baseline characteristics, initial therapy, or improvements of disease activity indicators between the resolved HBV group and the non-HBV group.

Spearman’s rank-order correlation test was used to assess the relationship between baseline serum HBV DNA levels and clinical outcomes in the CHB group. The results revealed positive correlations between HBV DNA titers and RAPID3 at month 1 (*r* = 0.671, *p* = 0.024) and month 3 (*r* = 0.713, *p* = 0.014). Significant correlations were also observed between a level of HBV DNA ≥ 10^4^ IU/ml and PtGA at month 12 (*r* = 0.645, *p* = 0.032), SDAI at month 6 (*r* = 0.635, *p* = 0.036), and RAPID3 at month 1 (*r* = 0.637, *p* = 0.035), month 6 (*r* = 0.637, *p* = 0.035), and month 12 (*r* = 0.638, *p* = 0.035).

### Radiographic progression

**Fig. 3:**
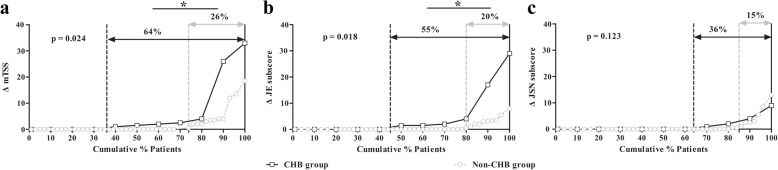
One-year radiographic changes of patients with rheumatoid arthritis (RA) with and without chronic hepatitis B virus infection (CHB). Comparison of cumulative probability of ∆mTSS (**a**), ∆JE subscore (**b**), and ∆JSN subscore (**c**) during 1-year follow-up between patients with RA with and without CHB. Cumulative probability distribution of radiographic change in mTSS from baseline to month 12 demonstrated that a significantly higher percentage of patients with CHB displayed 1-year radiographic progression. *p < 0.05. *JE* Joint erosion; *JSN* Joint space narrowing; *mTSS* Modified total Sharp score

No significant difference was found in JE subscore, JSN subscore, or mTSS between the CHB and non-CHB groups at baseline (all *p* > 0.05) (Table 1). Thirty-three percent of patients with RA had 1-year radiographic progression. Compared with the non-CHB group, a significantly higher percentage of patients with RA with CHB experienced 1-year radiographic progression (64% vs. 26%, *p* = 0.024), together with greater increases in JE subscore (1.5 [IQR 0–4.0] vs. 0 [IQR 0–0], *p* = 0.024) and mTSS (1.5 [IQR 0–4.0] vs. 0 [IQR 0–0.9], *p* = 0.024). The cumulative probability distribution of radiographic change in mTSS from baseline to month 12 for patients with RA in the CHB and non-CHB groups is shown in Fig. 3, where the space between the curves indicates that a higher percentage of patients with RA with CHB experienced 1-year radiographic progression. There were no significant differences in all these indicators between the resolved HBV group and the non-HBV group (all *p* > 0.05).

Spearman’s rank-order correlation test was used to assess the relationship between baseline serum HBV DNA levels and radiographic outcomes in the CHB group. The results revealed positive correlations between HBV DNA titers and increases in JSN subscore (*r* = 0.606, *p* = 0.048) and rapid radiographic progression (*r* = 0.677, *p* = 0.022).

### Risk factors for 1-year radiographic progression

To determine risk factors for 1-year radiographic progression, univariate logistic regression analysis was performed, including baseline characteristics and initial therapies after enrollment as variables. The results showed that CHB status, female sex, smoking status, and treatment-naïve status as well as higher baseline mTSS were significantly associated with 1-year radiographic progression (all *p* <  0.05) (Table 3). In bivariate analyses that were adjusted for the significant confounding factors in univariate logistic regression analysis, CHB status was always positively associated with 1-year radiographic progression (OR 4.632–7.069, all *p* <  0.05). Furthermore, multivariate logistic regression analysis that was adjusted for all significant factors in univariate analyses revealed that CHB status (OR 14.230, 95% CI 2.213–95.388, *p* = 0.006) and the count of CD68-positive macrophages (OR 1.002, 95% CI 1.001–1.003, *p* = 0.003) were independently associated with 1-year radiographic progression.

**Table 3:** Logistic regression analyses for risk factors of 1-year radiographic progression

Parameters	OR	95% CI	*p* Value^a^
Univariate analyses			
Female	0.258	(0.073–0.910)	**0.035**
Age	0.965	(0.922–1.010)	0.128
Disease duration	1.008	(1.000–1.017)	0.050
Smoking status	3.850	(1.024–14.473)	**0.046**
CHB status	4.958	(1.231–19.980)	**0.024**
TJC28	0..958	(0.885–1.126)	0.293
SJC28	1.017	(0.918–1.126)	0.752
PtGA	1.122	(0.853–1.476)	0.411
PrGA	1.031	(0.766–1.387)	0.841
Pain VAS	1.093	(0.824–1.448)	0.537
CRP	1.008	(0.991–1.025)	0.342
ESR	0.993	(0.997–1.010)	0.441
Positive RF	4.065	(0.462–35.752)	0.206
Positive ACPA	0.729	(0.111–4.778)	0.741
DAS28-CRP	0.975	(0.571–1.664)	0.926
HAQ-DI	0.724	(0.359–1.463)	0.369
mTSS	1.024	(1.004–1.044)	**0.021**
Treatment-naïve^b^	0.296	(0.094–0.935)	**0.038**
Glucocorticosteroids^c^	1.164	(0.307–4.412)	0.823
Methotrexate^c£^	1.000	(0.085–11.778)	0.999
Leflunomide^c^	0.397	(0.125–1.259)	0.117
Sulfasalazine^c^	1.905	(0.497–7.294)	0.347
Hydroxychloroquine^c^	3.111	(0.868–11.149)	0.081
Biological DMARDs^c^	0.357	(0.107–1.189)	0.093
MVCs	1.005	(0.996–1.014)	0.302
CD3-positive T cells^d^	1.000	(0.999–1.001)	0.982
CD15-positive neutrophils	1.001	(0.999–1.002)	0.393
CD20-positive B cells^e^	1.000	(1.000–1.001)	0.389
CD38-positive plasma cells^f^	1.000	(1.000–1.001)	0.396
CD68-positive macrophages	1.002	(1.000–1.003)	**0.006**
Bivariate models			
CHB status adjusted for gender	4.632	(1.089–19.697)	**0.038**
CHB status adjusted for smoking status	6.097	(1.397–26.616)	**0.016**
CHB status adjusted for treatment-naïve status	4.958	(1.231–19.980)	**0.024**
CHB status adjusted for baseline mTSS	7.069	(1.539–32.480)	**0.012**
Multivariate models^g^			
CHB status adjusted for gender, smoking status, treatment-naïve status, and baseline mTSS	14.230	(2.123–95.388)	**0.006**
CD68-positive macrophages adjusted for gender, smoking status, treatment-naïve status, and baseline mTSS	1.002	(1.001–1.003)	**0.003**

*Abbreviations: ACPA* Anti-cyclic citrullinated peptide antibody, *CHB* Chronic hepatitis B virus infection, *CRP* C-reactive protein, *DAS28* Disease Activity Score 28-joint assessment, *ESR* Erythrocyte sedimentation rate, *HAQ-DI* Stanford Health Assessment Questionnaire Disability Index, *mTSS* Modified total Sharp score, *MVC* Microvessel count, *Pain VAS* Pain visual analogue scale, *PrGA* Provider global assessment of disease activity, *PtGA* Patient global assessment of disease activity, *RA* Rheumatoid arthritis, *RF* Rheumatoid factor, *SJC28* 28-joint swollen joint count, *TJC28* 28-joint tender joint count

^a^Calculated using logistic regression analysis. Bold *p* values indicate statistically significant levels

^b^Without glucocorticosteroid or disease-modifying anti-rheumatic drug therapy since 6 months before enrollment

^c^Initial medications after enrollment

^£^Methotrexate: OR: 1.000000, 95% CI: 0.084904–11.778006, *p* = 0.999

^d^CD3-positive T cells: OR: 1.000011, 95% CI: 0.999047–1.000976, *p* = 0.982

^e^CD20-positive B cells: OR: 1.000272, 95% CI: 0.999653–1.000892, *p* = 0.389

^f^CD38-positive plasma cells: OR: 1.000261, 95% CI: 0.999658–1.000865, *p* = 0.396

^g^Owing to the multicollinearity between CHB status and the total count of CD68-positive macrophages, two multivariate models were established, respectively, by adjusting for all significant univariate factors

## Discussion

This study was performed using a prospective cohort of consecutive patients with RA with available synovium. The results of immunohistochemical staining and nested PCR revealed the presence of HBcAg and HBV *S* gene in the synovium from patients with RA with CHB. Of note, this is the first report, to our knowledge, of the presence of HBV in RA synovium. Synovial tissue is the primary target of disturbed immunomodulatory pathways in RA. Our previous study revealed HBV DNA in synovial fluid from patients with RA with CHB, but it failed to demonstrate positive HBsAg staining by immunohistochemistry in the synovium from patients with RA with either current or resolved HBV infection [7]. In the present study, even though results of HBsAg staining were negative using two different commercial antibodies against HBsAg, HBV was detected in the synovium of patients with RA with CHB, as evidenced by positive HBcAg immunoreactivity and further confirmation by nested PCR for the HBV *S* gene. HBcAg is a reliable marker for HBV infection and viral replication. Full-length HBc capsids could induce tumor necrosis factor-α (TNF-α), interleukin (IL)-6, and IL-12p40 via activation of nuclear factor kappa-B (NF-κB), extracellular signal-regulated protein kinases 1/2, and p38 mitogen-activated protein kinase in macrophages [28]. The distribution of HBcAg could be generally classified as cytoplasmic, nuclear, or mixed in expression [29]. Cytoplasmic HBcAg is more likely to be recognized by CD4^+^ T cells and acts as a target antigen of immune-mediated cytolysis, which implicates its role in the pathogenesis of liver damage caused by HBV infection [30, 31]. HBV DNA replicative intermediates and viral proteins can be detected in peripheral blood mononuclear cells (PBMCs) of patients with CHB, with monocytes and B cells being the most frequently infected cells [32]. Studies on vertical transmission revealed the presence of HBsAg and HBcAg in CD68^+^ cells of villous stroma and blood capillaries in placenta from mothers with HBV-positive PBMCs, which may serve as a vector for maternal-fetal transmission of HBV [33]. In our study, HBcAg was located mainly in the cytoplasm of plasma cells and sublining macrophages in the synovium, which may result partially from migration and differentiation of HBV-infected PBMCs. However, studies have suggested that the severity of extrahepatic disease in patients with HBV infection might be related to viral burden, which may need to reach a certain threshold before extrahepatic HBV syndromes become clinically evident [5, 34]. In this study, higher baseline serum HBV DNA levels were observed to be positively correlated with poorer clinical and radiographic outcomes, indicating that higher levels of HBV DNA may contribute to more pronounced disease progression. However, our results showed that not all patients with RA with CHB had HBV markers in the synovium and that the intensity of HBcAg expression was not completely in line with serum HBV DNA level. Owing to confounding factors such as different antiviral therapies and anti-RA regimens among different patients, it may not suffice to simply investigate the relationship between serum HBV DNA levels and RA clinical characteristics. Future studies should feature larger numbers of patients with RA with CHB and thus provide sufficient statistical power for further multivariate logistic regression analyses.

Further analyses of the influence of HBV infection on histopathological characteristics of synovitis showed more pronounced CD68-positive macrophages, CD20-positive B cells, and CD15-positive neutrophils infiltrating CHB synovium. Despite the small number of CHB specimens, this group seemed to have a higher synovial stroma activation subscore, more MVCs, and more pronounced sublining CD68-positive macrophages as well as CD20-positive B cells in CHB synovium with positive HBcAg than without it. Synovial macrophages are the main source of proinflammatory cytokines, including TNF-α and IL-1. Their density (cell count per unit area) is associated with synovial inflammation and joint destruction in RA, and it has a predictive role in evaluating the clinical efficacy of RA treatment [35]. Increased CD20-positive B cells infiltrating RA synovium could promote disease progression by producing autoantibodies and cytokines such as TNF-α, IL-6, and receptor activator of NF-κB ligand [36], enhancing osteoclastogenesis. B-cell-targeted therapy such as rituximab can alleviate such abnormalities and improve disease prognosis. Neutrophils have been a focus of RA research since the discovery of neutrophil extracellular traps (NETs), and increased components of NETs have been found in RA sera [37]. NETs are highly enriched in specific autoantigens such as citrullinated proteins targeted by ACPA in patients with RA [38, 39], but they also provide stimuli to fibroblast-like synoviocytes [40], dendritic cells [41], macrophages [42], and lymphocytes [43, 44], which promote systemic and local (synovial) autoimmune responses. However, although no significant difference was found in CD3^+^ T-cell count between CHB synovium with and without positive HBcAg, there was a trend of more CD3^+^ T cells infiltrating CHB synovium than non-CHB synovium (1141 [560–1751]/mm^2^ vs. 639 [473–1131]/mm^2^, *p* = 0.124). The small number of RA synovial tissues may have precluded us from obtaining a statistically significant difference. In the present study, we only used CD3 to stain for T cells in RA synovium. Therefore, we cannot rule out the possibility of an increased count or an enhanced activity of some T-cell subsets in the CHB synovium. Further explorations on the topic and the potential mechanism are needed. In total, HBV infection—especially its presence in synovium—may play a role in the pathogenesis of local lesions of synovitis in RA.

Previous studies have indicated that HBV infection was more likely to be an exacerbating factor in the pathogenicity and progression of RA. Arthritis of several patients with HBV infection who fulfilled the ACR diagnostic criteria for RA could be resolved by anti-HBV therapy [45, 46]. An acute case of seropositive RA was reported in a woman 24 hours after receiving the first dose of hepatitis B vaccine. She showed a steady improvement after receiving glucocorticosteroids and sulfasalazine, but x-rays of both hands showed erosions with minimal periarticular osteoporosis 10 months later [47]. In the present study, analysis of the influence of HBV infection on clinical and radiographic outcomes showed smaller improvements from baseline in most disease activity indicators at month 12, with a significantly higher percentage of patients with CHB experiencing 1-year radiographic progression, and multivariate logistic regression analysis revealed that CHB status was an independent risk factor for 1-year radiographic progression in RA. These results were consistent with the aforementioned hypothesis and further implied that HBV infection might exacerbate disease progression, causing poor clinical response and subsequent radiographic progression in patients with RA with CHB. With the results of histopathological characteristics of synovitis, we speculated that RA concurrent with HBV infection, especially its presence in synovium, may be classified as a new phenotype of HBV-induced RA that may need adjusted and specific treatment regimens to obtain a satisfactory therapeutic response.

There are several limitations of this study. First, the small number of patients with RA with CHB and synovial tissue of adequate quality clearly limits its statistical power. There was no significant difference in disease activity or 1-year radiographic progression between patients with CHB with and without HBcAg expression in synovium. Despite the small number of CHB synovial samples, patients with positive HBcAg in synovium seemed to have more pronounced synovitis than those without it. Second, compared with the non-CHB group, a tendency of longer disease duration (120 [6–120] vs. 24 [7–72], *p* = 0.345) and a smaller percentage of medication-naïve patients (46% vs. 65%, *p* = 0.226) were observed in the CHB group, which may lead to significantly lower levels of most disease activity indicators at baseline and subsequent analyses at each visit. Further prospective cohort studies of RA with more patients with CHB, especially treatment-naïve patients with early disease, are needed. The potential mechanism of HBV infection in RA progression, the possibility of a new phenotype of HBV-induced RA, and the method of choosing DMARDs and antiviral therapy for these patients merit further exploration.

## Conclusions

This study reveals the presence of HBV in the synovium of patients with RA with CHB. HBV may be involved in the pathogenesis of local lesions and exacerbate disease progression, including disease activity and joint destruction.

## Additional file

Additional file 1: Dynamic disease activity indicators in all patients with RA during 1-year follow-up^†^. (PDF 71 kb)

## Abbreviations

ACPA: Anti-cyclic citrullinated peptide antibody
ACR: American College of Rheumatology
ALT: Alanine aminotransferase
AST: Aspartate transaminase
CDAI: Clinical Disease Activity Index
CHB: Chronic hepatitis B virus infection
CRP: C-reactive protein
DAS28: Disease Activity Score 28-joint assessment
DMARD: Disease-modifying anti-rheumatic drug
ESR: Erythrocyte sedimentation rate
EULAR: European League Against Rheumatism
HAQ-DI: Stanford Health Assessment Questionnaire Disability Index
HBcAg: Hepatitis B virus core antigen
HBeAg: Hepatitis B virus e antigen
HBsAg: Hepatitis B virus surface antigen
HBV: Hepatitis B virus
ICC: Intraclass correlation coefficient
IL: Interleukin
IQR: Interquartile range
JE: Joint erosion
JSN: Joint space narrowing
mTSS: Modified total Sharp score
MVC: Microvessel count
NA: Not applicable
NET: Neutrophil extracellular trap
NF-κB: Nuclear factor-κB
PBMC: Peripheral blood mononuclear cell
PrGA: Provider global assessment of disease activity
PtGA: Patient global assessment of disease activity
RA: Rheumatoid arthritis
RAPID3: Routine Assessment of Patient Index Data 3
RF: Rheumatoid factor
SDAI: Simplified Disease Activity Index
SJC28: 28-joint swollen joint count
TJC28: 28-joint tender joint count
TNF-α: Tumor necrosis factor-α
VAS: Visual analogue scale
