# Supplementary material for: Presence of hepatitis B virus in synovium and its clinical significance in rheumatoid arthritis
Source: Arthritis Res Ther. 2018 Jun 19;20:130. doi: 10.1186/s13075-018-1623-y (PMC6009044; doi:10.1186/s13075-018-1623-y)
Supplement: Supplementary file 1 — Dynamic disease activity indicators in all patients with RA during 1-year follow-up†. (PDF 71 kb) [file 13075_2018_1623_MOESM1_ESM.pdf]

Additional file 1 Dynamic disease activity indicators in all RA patients during one-year follow-up†

Disease activity indicators	1st month	3rd month	6th month	12th month
TJC28	4 (1-9)***	2 (0-5)***	1 (0-4)***	1 (0-4)***
SJC28	2 (0-5)***	1 (0-2)***	0 (0-1)***	0 (0-2)***
PtGA	3 (2-5)***	3 (1-4)***	2 (0-4)***	2 (0-4)***
PrGA	3 (1-5)***	2 (1-4)***	2 (0-4)***	2 (0-4)***
PainVAS	2.0 (1.0-4.0)***	2.0 (0.5-3.5)***	2.0 (0-3.0)***	2.0 (0-3.0)***
CRP, mg/l	7.7 (3.4-23.3)***	7.7 (3.3-16.0)***	4.2 (3.3-10.3)***	4.8 (3.1-9.0)***
ESR, mm/h	35 (20-56)***	30 (18-45)***	29 (20-43)***	30 (17-40)***
DAS28-CRP	3.8 (2.5-5.0)***	3.1 (2.1-4.1)***	2.7 (1.8-3.5)***	2.6 (1.7-3.6)***
DAS28-ESR	4.0 (3.0-5.7)***	3.5 (2.7-4.9)***	3.4 (2.5-4.3)***	3.4 (2.4-4.0)***
SDAI	14.2 (5.3-27.4)***	9.3 (3.6-18.1)***	7.3 (2.3-15.7)***	6.3 (0.8-14.1)***
CDAI	12 (5-24)***	9 (2-16)***	6 (2-15)***	6 (0-12)***
RAPID3	5.5 (3.0-9.1)***	4.1 (1.4-8.5)***	4.0 (1.0-7.1)***	3.0 (0-6.6)***
HAQ-DI	0.33 (0-0.98)***	0.15 (0-0.50)***	0.05 (0-0.38)***	0.13 (0-0.37)***

†Compared between baseline and follow-up each visit by Wilcoxon matched-pairs signed ranks sum test. Data correspond to n (percentage) or median (interquartile range) unless stated otherwise.

*p<0.05, **p<0.01, ***p<0.001.

Abbreviations: CDAI: Clinical disease activity index; CRP: C-reactive protein; DAS28: Disease Activity Score 28-joint assessment; ESR: Erythrocyte sedimentation rate; HAQ-DI: Chinese-language version of the Stanford Health Assessment Questionnaire Disability Index; Pain VAS: Pain visual analogue scale; PrGA: Provider global assessment of disease activity; PtGA: Patient global assessment of disease activity; RAPID3: Routine Assessment of Patient Index Data 3; SDAI: Simplified disease activity index; SJC28: 28-joint swollen joint counts; TJC28: 28-joint tender joint counts.
